# HIF-1α mediates hypertension and vascular remodeling in sleep apnea via hippo–YAP pathway activation

**DOI:** 10.1186/s10020-024-00987-5

**Published:** 2024-12-28

**Authors:** Shoude Zhang, Yuan Zhao, Zhanwei Dong, Mao Jin, Ying Lu, Mina Xu, Hong Pan, Guojin Zhou, Mang Xiao

**Affiliations:** 1 Department of Otorhinolaryngology/Head and Neck, Sir Run Run Shaw Hospital, Zhejiang University School of Medicine, No.3 East Qingchun Road, Hangzhou, 310020 Zhejiang China; 2 Department of Otorhinolaryngology/Head and Neck, Aral Hospital, Xinjiang Corps, Sir Run Run Shaw Hospital, Zhejiang University School of Medicine, Aral, 843399 Xinjiang China; 3 Department of Otorhinolaryngology/Head and Neck, The First People’s Hospital of Lin’an District, Hangzhou, 311300 Zhejiang China; 4 Department of Nursing, Sir Run Run Shaw Hospital, Zhejiang University School of Medicine, Hangzhou, 310020 Zhejiang China

**Keywords:** Sleep apnea syndrome, HIF-1α, Hippo, YAP, Vascular remodeling, Inflammation

## Abstract

**Background:**

Sleep apnea syndrome (SAS) is associated with hypertension and vascular remodeling. Hypoxia-inducible factor-1α (HIF-1α) and the Hippo–YAP pathway are implicated in these processes, but their specific roles remain unclear. This study investigated the HIF-1α/Hippo-YAP pathway in SAS-related hypertension.

**Methods:**

We established a rat model of SAS-induced hypertension via chronic intermittent hypoxia (CIH). Rats were treated with siRNA targeting HIF-1α. Blood pressure, inflammation, oxidative stress, vascular remodeling, and VSMC function were assessed. In vitro experiments with A7r5 cells and human aortic smooth muscle cells (HAoSMCs) explored the effects of HIF-1α silencing and YAP1 overexpression.

**Results:**

Compared with the control group, the CIH group presented significant increases in both HIF-1α and YAP1 expression, which correlated with increased blood pressure and vascular changes. HIF-1α silencing reduced hypertension, oxidative stress, inflammation, and the severity of vascular remodeling. Specifically, siRNA treatment for HIF-1α normalized blood pressure, decreased the levels of oxidative damage markers (increased SOD and decreased MDA), and reversed the changes in the levels of inflammatory markers (decreased high-sensitivity C-reactive protein (hs-CRP), interleukin-6 (IL-6) and soluble E-selectin (sE-s)). Structural analyses revealed reduced vascular smooth muscle cell proliferation and collagen deposition, along with normalization of cellular markers, such as α-SMA and TGF-β1. Furthermore, the Hippo–YAP pathway appeared to mediate these effects, as evidenced by altered YAP1 expression and activity upon HIF-1α modulation.

**Conclusions:**

Our findings demonstrate the significance of the HIF-1α/Hippo-YAP pathway in CIH-induced hypertension and vascular remodeling. HIF-1α contributes to these pathophysiological processes by promoting oxidative stress, inflammation, and aberrant VSMC behavior. Targeting this pathway could offer new therapeutic strategies for CIH-related cardiovascular complications in SAS patients.

**Supplementary Information:**

The online version contains supplementary material available at 10.1186/s10020-024-00987-5.

## Introduction

Sleep apnea syndrome (SAS) refers to the number of repeated apnea and hypopnea events over thirty during 7 h of sleep per night or a sleep apnea hypopnea index ≥ 5 times/h (Bhadriraju et al. 2008). Chronic intermittent hypoxia (CIH), which is related to repeated upper airway collapse during sleep in SAS patients, is the major physiological feature of SAS and the main stimulus for comorbidities of SAS, such as cardiovascular comorbidities and systemic hypertension (Ma et al. 2016; Jordan et al. 2014). Numerous studies have revealed that SAS-induced CIH is an independent risk factor for hypertension (Seravalle and Grassi 2022; Wang et al. 2016), and the incidence of hypertension is significantly elevated in SAS patients (Akashiba et al. 2022). SAS causes or exacerbates hypertension via acute physiological impacts such as elevated left ventricular afterload, decreased left ventricular preload, increased sympathetic nerve activity, and increased neurohumoral factors (Floras 2015). Vascular remodeling has been identified as the basis of the pathogenesis of SAS with hypertension (Arnaud et al. 2020). Therefore, investigating the underlying mechanism of CIH-induced hypertension might provide novel therapeutic targets for clinical practice.

Emerging evidence suggests that alterations in the transcription of hypoxia-inducible factors (HIFs) constitute a crucial molecular mechanism of SAS in hypertension (Prabhakar et al. 2020). HIF-1 was the first discovered part of the HIF family, followed by HIF-2 (Heer et al. 2020). HIF-1 is present in all mammalian cells, but HIF-2 is limited to certain tissues, including developing blood vessels, the lung, and the adrenal medulla (Zhang and Kong 2023). Both HIF-1 and HIF-2 can be composed of an O_2_-regulated α subunit together with a constitutive β subunit (Ceranski et al. 2023). Hypoxia-inducible factor‐1α (HIF‐1α) is a subunit of HIF‐1 that plays a vital role in hypoxia-related signaling pathways by modulating various cellular and molecular events (Li et al. 2019). HIF‐1α has been shown to be highly expressed in patients with SAS and hypertension (Lu et al. 2017). However, the specific role and possible mechanism of HIF‐1α in the SAS in patients with hypertension are obscure.

The Hippo–Yes–associated protein (YAP) pathway affects many diseases (Lv and Ai 2022). YAP1 is a transcriptional activator of Hippo signaling that combines with the HIF-1α protein in the nucleus and maintains its stability (Kashihara et al. 2022). As reported previously, the Hippo–YAP pathway is implicated in promoting inflammation and oxidative stress in diseases (Zheng et al. 2021; Shao et al. 2014). In addition, the Hippo–YAP pathway can change the production of the extracellular matrix as well as vascular smooth muscle cell growth and migration, contributing to vascular remodeling (He et al. 2018). However, the potential of the Hippo–YAP pathway in SAS-related hypertension is unclear.

Therefore, this study explored the potential of the HIF-1α/Hippo-YAP pathway in the progression of SAS-related hypertension.

## Materials and methods

### Construction of the animal model

The animal experiments were implemented with the approval of the animal ethical committee of our hospital. Eight-week-old male Sprague‒Dawley rats (Vital River, Beijing, China) were housed in a specific pathogen-free room at the proper temperature and provided with adequate food and water. Rats were exposed to CIH via an anoxic apparatus to establish an animal model of SAS complicated with hypertension. The specific procedure was as follows: (a) nitrogen (7%) was added for 1 min to maintain hypoxia, and (b) oxygen (20%) was added for 0.5 min to maintain reoxygenation. The cycles of a and b could be repeated every day from 9 am to 5 pm, and the total experiments lasted for 56 days. The rats were not given food or water when exposed to CIH via a hypoxic instrument. The control rats were kept in the same apparatus under normal air conditions (He et al. 2020; Guo et al. 2022). The blood pressure of the caudal artery was measured via a blood pressure monitor on days 0, 14, 28, 42, and 56. At the end of the experiment, an animal model with a blood pressure greater than 150 mmHg was successfully established.

### In vivo HIF-1α treatment

The rats were randomly separated into (a) control, (b) CIH, and (c) CIH + siRNA-HIF-1α groups (*n* = 6 per group). The recombinant adeno-associated virus (rAAV) vector was used as a negative control (NC). The rAAV vector carrying siRNA-HIF-1α and the control vector were obtained from Hanbio (Shanghai, China). Rats in the CIH + HIF-1α group were injected daily with the rAAV vector (1 × 10^11^ vg/100 µL) carrying siRNA-HIF-1α via the tail vein prior to CIH exposure (Guo et al. 2022). Fifty-six days later, the rats were euthanized, and blood, heart tissue, and the abdominal aorta were collected.

### RT‒qPCR

Total RNA was extracted with TRIzol reagent (Life Technologies) and reverse transcribed with a 5 × All-In-One kit (Applied Biological Materials, Canada). Afterward, gene expression was examined with SYBR Green Master Mix (Applied Biosystems, USA). GAPDH was used as an endogenous control, and gene expression was calculated via the 2^−∆∆CT^ method.

### Immunohistochemistry

In brief, 4‑µm paraffin-embedded sections were subjected to deparaffinization and heat-induced antigen recovery. Next, the samples were blocked with QuickBlock Blocking Buffer (Beyotime) for 30 min, followed by incubation with primary antibodies containing anti-α-SMA and anti-TGF-β1 at 4 °C overnight. The secondary antibodies were then added for cultivation at 37 °C for 1 h. DAPI (Beyotime) was used to counterstain the nuclei. Images were obtained with a microscope (Rueil-Malmaison).

### Terminal deoxynucleotidyl transferase UTP nick end labeling (TUNEL)

Apoptosis in rat heart and aortic tissues was analyzed via TUNEL assay kit (Roche Applied Science, Switzerland) following the manufacturer’s instructions. Briefly, tissue sections were prepared, and the sections were incubated with 50 µL of TUNEL mixture for 60 min at 37 °C in the dark. The sections were subsequently washed with PBS and stained with DAPI. The TUNEL-positive cells were observed under a Nikon fluorescence microscope (Ti2-U, Tokyo, Japan).

### Western blot

The rat tissues were removed and placed in a 2.0 mL centrifuge tube, followed by treatment with 1 mL of RIPA lysis buffer containing 10 µL of PMSF protease inhibitor (Beyotime, China). A Nuclear and Cytoplasmic Protein Extraction Kit (Beyotime, China) was used to isolate the proteins from the cytoplasm and nuclei, respectively. The mixture was subsequently homogenized on ice. After centrifugation, the liquid layer containing the total protein was transferred to a new tube. After the protein concentration was determined, the total protein was separated via SDS‒PAGE and then transferred to polyvinylidene difluoride (PVDF) membranes (Millipore, USA). Afterwards, the membranes were blocked for 2 h with 5% skim milk powder, followed by incubation with primary antibodies, including anti-HIF-1α (1:1000, ab179483, Abcam), anti-TGF-β1 (1:1000, ab215715, Abcam), anti-α-SMA (1:10000, ab124964, Abcam), anti-Ki67 (1:100, ab231172, Abcam), anti-Bax (1:1000, ab32503, Abcam), and anti-Bcl-2 (1:1000, ab194583, Abcam), for 12 h as well as secondary antibodies (1:2000, ab6721, Abcam) for 1.5 h. The protein bands were detected via chemiluminescence reagents (Pierce, USA).

### Enzyme-linked immunosorbent assay (ELISA)

The collected blood was centrifuged to isolate the serum. The serum levels of superoxide dismutase (SOD, #MBS266897), malondialdehyde (MDA, #MBS268427), high-sensitivity C-reactive protein (hs-CRP, #MBS764381), interleukin-6 (IL-6, # MBS2020158) and soluble E-selectin (sE-s, #MBS2533566) were determined with commercial ELISA kits provided by MyBioSource (San Diego, CA, USA).

### Histopathological analysis

After fixation with 4% formaldehyde, the sections were dehydrated, embedded, and sliced into 5 μm thick sections. Hematoxylin and eosin (H&E; Sigma‒Aldrich, USA) staining and Masson’s trichrome staining (Maixin Biotechnology, China) were carried out following the manufacturer’s instructions. The Masson-stained positive areas were quantified by ImageJ software to evaluate fibrosis.

### Cell culture and transfection

Rat aortic smooth muscle cells (A7r5) purchased from the Chinese Academy of Sciences (Shanghai, China) and human aortic smooth muscle cells (HAoSMCs) purchased from American Type Culture Collection (ATCC, MA, USA) were incubated in DMEM (Gibco, USA) supplemented with 10% fetal bovine serum (Gibco, USA) at 37 °C with 5% CO_2_. Two specific siRNAs targeting HIF-1α (siRNA-HIF-1α#1 and siRNA-HIF-1α#2) and the negative control (siRNA-NC) and a YAP1 overexpression vector (oe-YAP1) and negative control (oe-NC) were purchased from RiboBio (Guangzhou, China). The cells were transfected via Lipofectamine 3000 (Invitrogen, USA) under hypoxic conditions (5% CO_2_, 1% O_2_, and 94% N_2_).

### EdU

The cells were plated into 48-well plates, and cell proliferation was detected via a Cell-Light EdU Apollo 567 In Vitro Kit (RiboBio, China). The cells were treated with EdU solution (50 µM) for 2 h at 37 °C and then processed following the manufacturer’s instructions. Images of the cells were acquired under a microscope (Olympus, Japan).

### Colony formation assay

Briefly, 2000 cells were plated into 6-well plates for culture. The cells were grown for 10 days, followed by crystal violet staining (Solarbio, Beijing, China). The number of colonies was manually counted.

### Flow cytometry

The cells were cultivated in 6-well plates after transfection. After 48 h, cell apoptosis was determined via a FITC Annexin V Apoptosis Detection Kit (BD Biosciences, USA), followed by evaluation via a flow cytometer (BD Biosciences, USA).

### Transwell

The cells were seeded in the upper chamber of a Transwell plate. The lower chamber contained medium supplemented with 10% FBS. After 12 h, the nonmigrated cells were removed, and the migrated cells were fixed and stained with crystal violet. The cells were photographed under a microscope.

### Statistical analysis

Each experiment was performed three times. SPSS 22.0 software (IBM Company, USA) was used for the data analysis. The differences were analyzed by t tests or one-way ANOVA. The data are shown as the means ± SDs. *P* < 0.05 indicated statistical significance.

## Results

### HIF-1α and YAP1 are overexpressed in a rat model of SAS-Induced Hypertension

**Fig. 1:**
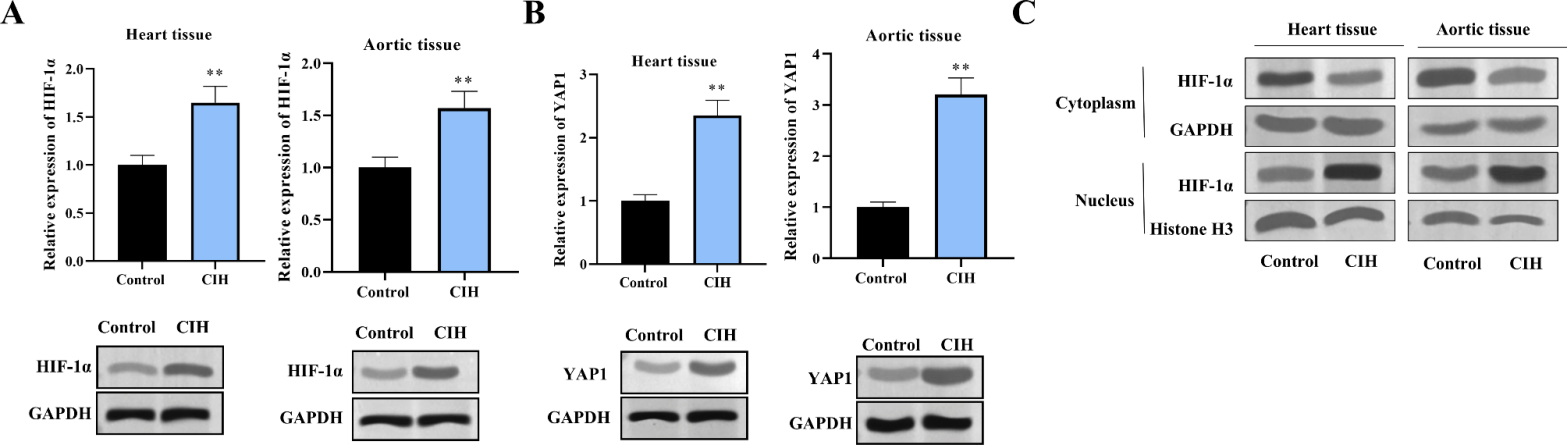
Elevated HIF-1α and YAP1 expression in a rat model of SAS-induced hypertension. (**A-B**) RT‒qPCR and western blot analysis revealed significantly increased expression of HIF-1α and its downstream target YAP1 in both the heart and aortic tissues of rats exposed to chronic intermittent hypoxia (CIH) compared with those of controls. *n* = 6 per group. (**C**) Western blotting was used to detect the expression of HIF-1α in the cytoplasm and nucleus of rat heart and aortic tissues. The data are shown as the means ± SDs. (**P < 0.01)

We first established that the expression of HIF-1α and its downstream target YAP1 was increased in a rat model of CIH, a hallmark of SAS. Using RT‒qPCR and western blot techniques, we demonstrated elevated levels of both HIF-1α and YAP1 in the cardiac and aortic tissues of CIH-exposed rats than in those of control rats (Fig. 1A‒B). Additionally, we detected the cytoplasmic and nuclear expression of HIF-1α in heart tissue and aortic tissue. The results showed that CIH induced the transportation of HIF-1α from the cytoplasm to the nucleus, which indicated that CIH promoted the activation of HIF-1α in vivo (Fig. 1C). These findings provide a molecular link between SAS and the cardiovascular pathologies frequently observed in SAS.

### Silencing HIF-1α attenuates hypertension, oxidative stress, and inflammation

**Fig. 2:**
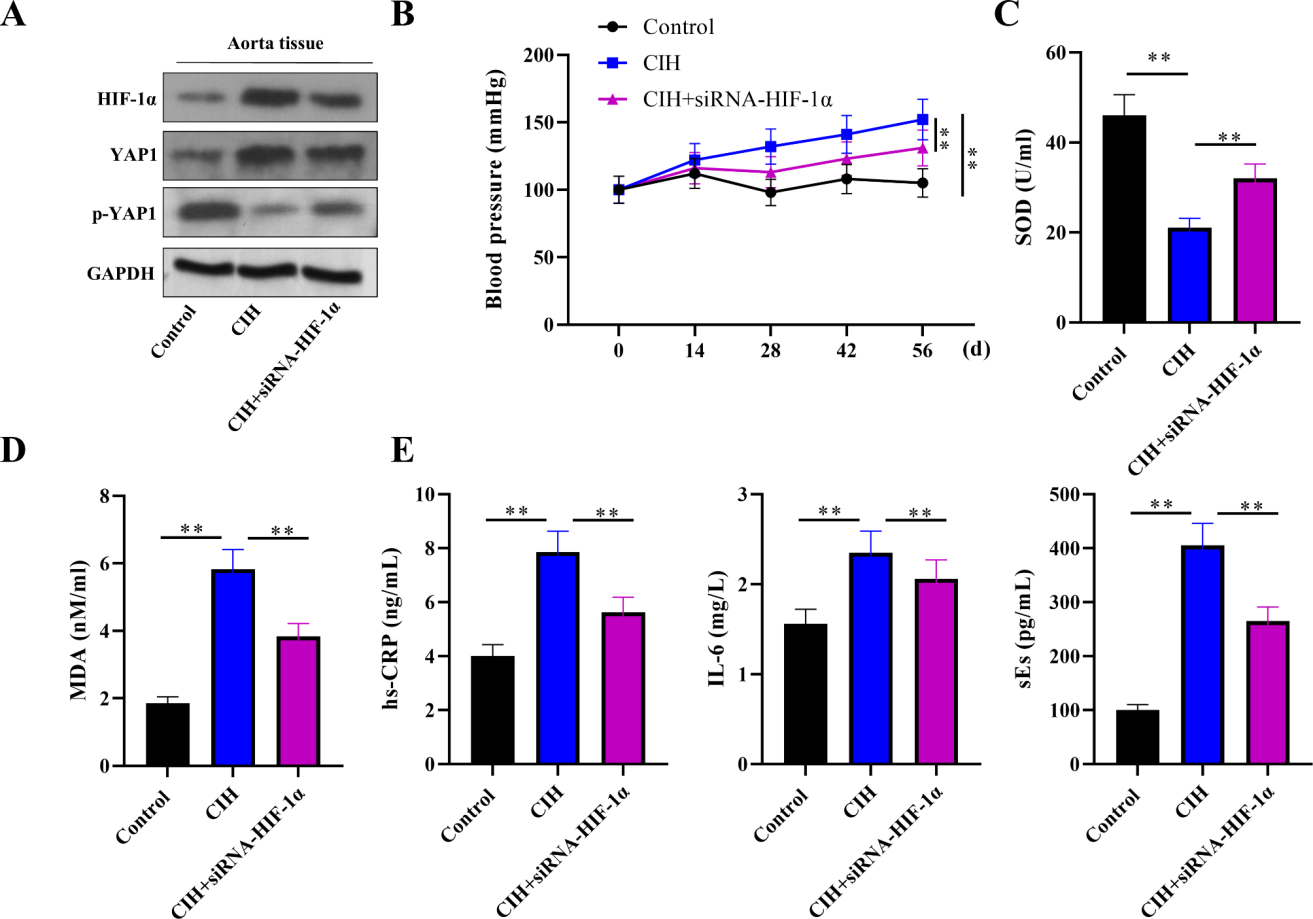
HIF-1α knockdown attenuates hypertension, oxidative stress, and inflammation in SAS. (**A**) Western blotting was performed to detect the protein expression of HIF-1α, YAP1, and p-YAP1 in the aortic tissues of rats in the control, CIH and CIH + siRNA-HIF-1α groups. (**B**) Systolic blood pressure measurements over a 56-day CIH exposure period. Compared with those in the CIH-only group, the blood pressure of the rats pretreated with siRNA-HIF-1α was significantly lower. (**C-D**) ELISA analysis revealed decreased superoxide dismutase (SOD) activity and increased malondialdehyde (MDA) levels, indicative of oxidative stress, in the CIH group. HIF-1α silencing rescues SOD activity and reduces MDA. (**E**) Elevated levels of inflammatory markers, including high-sensitivity C-reactive protein (hs-CRP), interleukin-6 (IL-6), and soluble E-selectin (s-Es), were detected in the CIH group. HIF-1α depletion significantly normalizes these inflammatory mediators. *n* = 6 per group. The data are shown as the means ± SDs. (**P < 0.01)

To elucidate the functional role of HIF-1α in the pathogenesis of hypertension associated with SAS, we administered siRNA targeting HIF-1α to rats prior to exposure to CIH conditions. The knockdown efficiency of HIF-1α was validated in the aorta tissues of CIH rats. We also demonstrated that the downstream target YAP1 was upregulated and that its phosphorylation level (p-YAP1) was reduced in the aortic tissues of CIH rats, whereas HIF-1α silencing decreased the expression of YAP1 and elevated p-YAP1 levels, suggesting that the activation of the Hippo–YAP signaling pathway in CIH rats was suppressed by HIF-1α knockdown (Fig. 2A). Figure 2B shows a progressive increase in systolic blood pressure over 56 days in the CIH-exposed rats. Conversely, rats in the CIH + siRNA-HIF-1α group displayed a notable reduction in blood pressure, underscoring the hypertensive role of HIF-1α in this model.

Furthermore, we investigated the influence of HIF-1α on oxidative stress and inflammation, which are critical components in the development of hypertension in SAS. Our data, presented in Fig. 2C, indicate that superoxide dismutase (SOD) activity was significantly compromised in the CIH group but was preserved upon HIF-1α silencing, suggesting a protective role against oxidative damage. Additionally, the levels of malondialdehyde (MDA), a marker of lipid peroxidation, were elevated in the CIH group but were mitigated by siRNA-HIF-1α treatment (Fig. 2D).

Inflammatory profiling further revealed elevated levels of high-sensitivity C-reactive protein (hs-CRP), interleukin-6 (IL-6), and serum endothelin (s-Es) in the CIH group. Notably, these inflammatory markers were substantially reversed following the depletion of HIF-1α (Fig. 2E). These findings suggest that HIF-1α not only contributes to the hypertensive response but also modulates oxidative stress and inflammation in this rat model of SAS-induced hypertension.

### Silencing HIF-1α attenuates SAS-Induced Vascular Remodeling

**Fig. 3:**
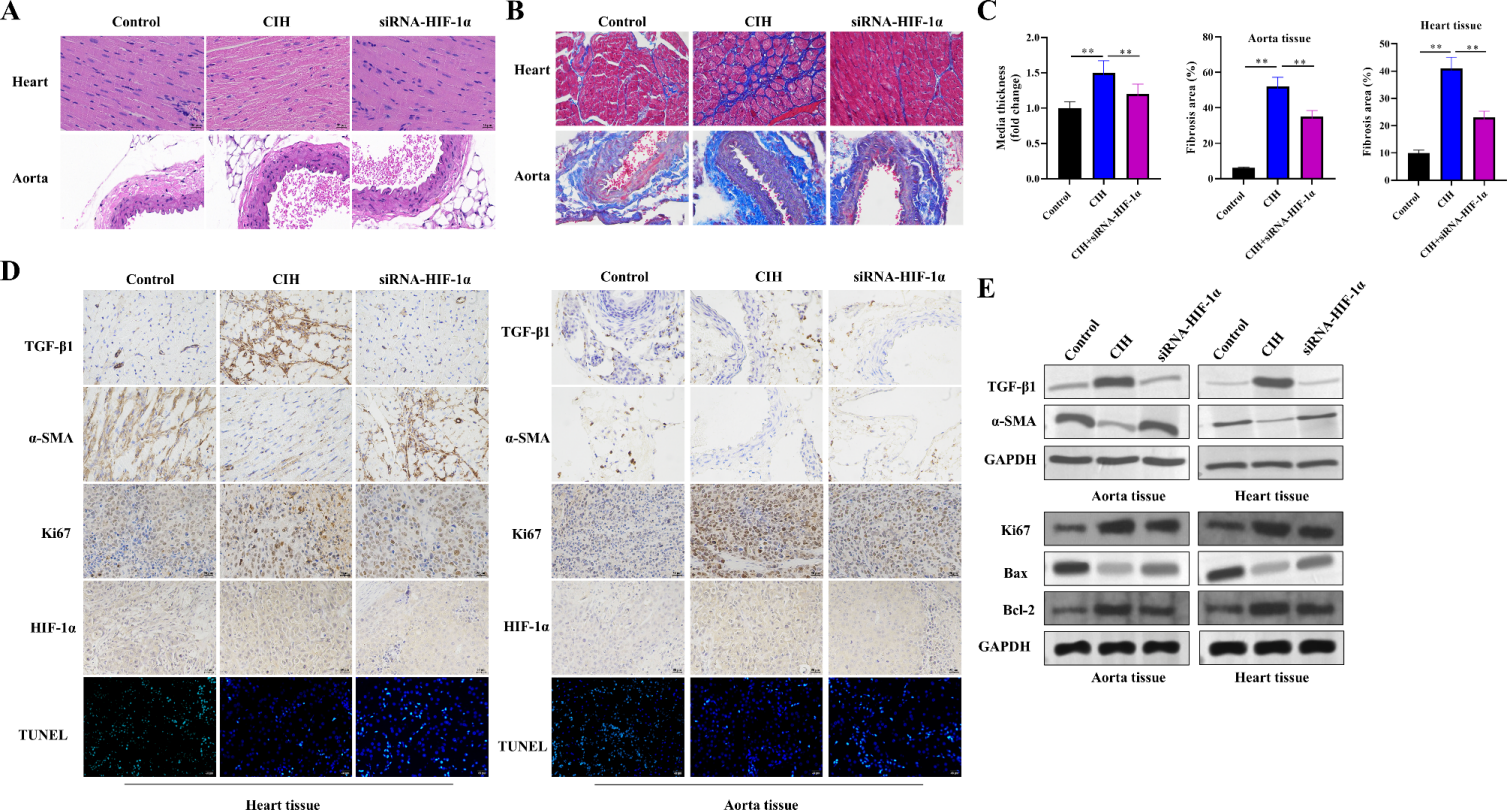
silencing HIF-1α mitigates SAS-induced vascular remodeling. (**A**) Representative H&E staining of heart and abdominal aorta sections. CIH exposure leads to cardiomyocyte degeneration, blood vessel dilation, irregular arrangement of vascular smooth muscle cells (VSMCs), and increased medial thickness. These effects are attenuated by siRNA-HIF-1α treatment. (**B**) Masson’s trichrome staining highlights increased collagen deposition (blue) in CIH-exposed rats, particularly within the vessel wall. siRNA-HIF-1α administration diminishes collagen accumulation. (**C**) Quantitative analysis of media wall thickness and fibrotic areas following HIF-1α silencing. (**D**) Immunohistochemical staining revealed decreased α-smooth muscle actin (α-SMA) and increased transforming growth factor-β1 (TGF-β1), Ki67 and HIF-1α expression in CIH-exposed hearts and aortas. siRNA-HIF-1α treatment reversed these changes. TUNEL assays were used to detect apoptosis in the rat heart and aorta. (**E**) Western blot analysis revealed downregulation of α-SMA and Bax and upregulation of TGF-β1, Ki67, and Bcl-2 in the CIH group, with restoration upon HIF-1α knockdown. *n* = 6 per group. The data are shown as the means ± SDs. (**P < 0.01)

We investigated the effects of HIF-1α silencing on structural alterations characteristic of vascular remodeling. Histological analysis via H&E staining revealed cardiomyocyte degeneration and blood vessel dilation in the CIH group. Importantly, these pathological changes were significantly reduced in the rats pretreated with siRNA-HIF-1α. Additionally, the CIH group exhibited an irregular arrangement of vascular smooth muscle cells (VSMCs) and increased thickness of the vascular media layer, both of which were ameliorated following siRNA-HIF-1α treatment. Furthermore, Masson’s trichrome staining revealed increased collagen deposition within the vessel wall in the CIH group, indicative of vascular fibrosis; this effect was also mitigated following HIF-1α knockdown (Fig. 3A‒C). To characterize vascular remodeling further, we assessed the expression of α-smooth muscle actin (α-SMA), a marker of the VSMC contractile phenotype, and transforming growth factor-β1 (TGF-β1), a potent profibrotic factor. Immunohistochemistry and western blot analyses revealed decreased α-SMA and increased TGF-β1 expression in the CIH group. Additionally, we verified the CIH-induced upregulation of HIF-1α and Ki67 and the reduction in TUNEL-positive cells. Western blot analysis also revealed that the expression of Ki67 and Bcl-2 was elevated, whereas that of Bax was decreased in the rat aorta tissue and heart tissue in the CIH group. Strikingly, these alterations were reversed following siRNA-HIF-1α administration (Fig. 3D and E), suggesting that HIF-1α plays a key role in promoting the phenotypic changes associated with vascular remodeling.

### HIF-1α promotes VSMC Proliferation and Migration while suppressing apoptosis

**Fig. 4:**
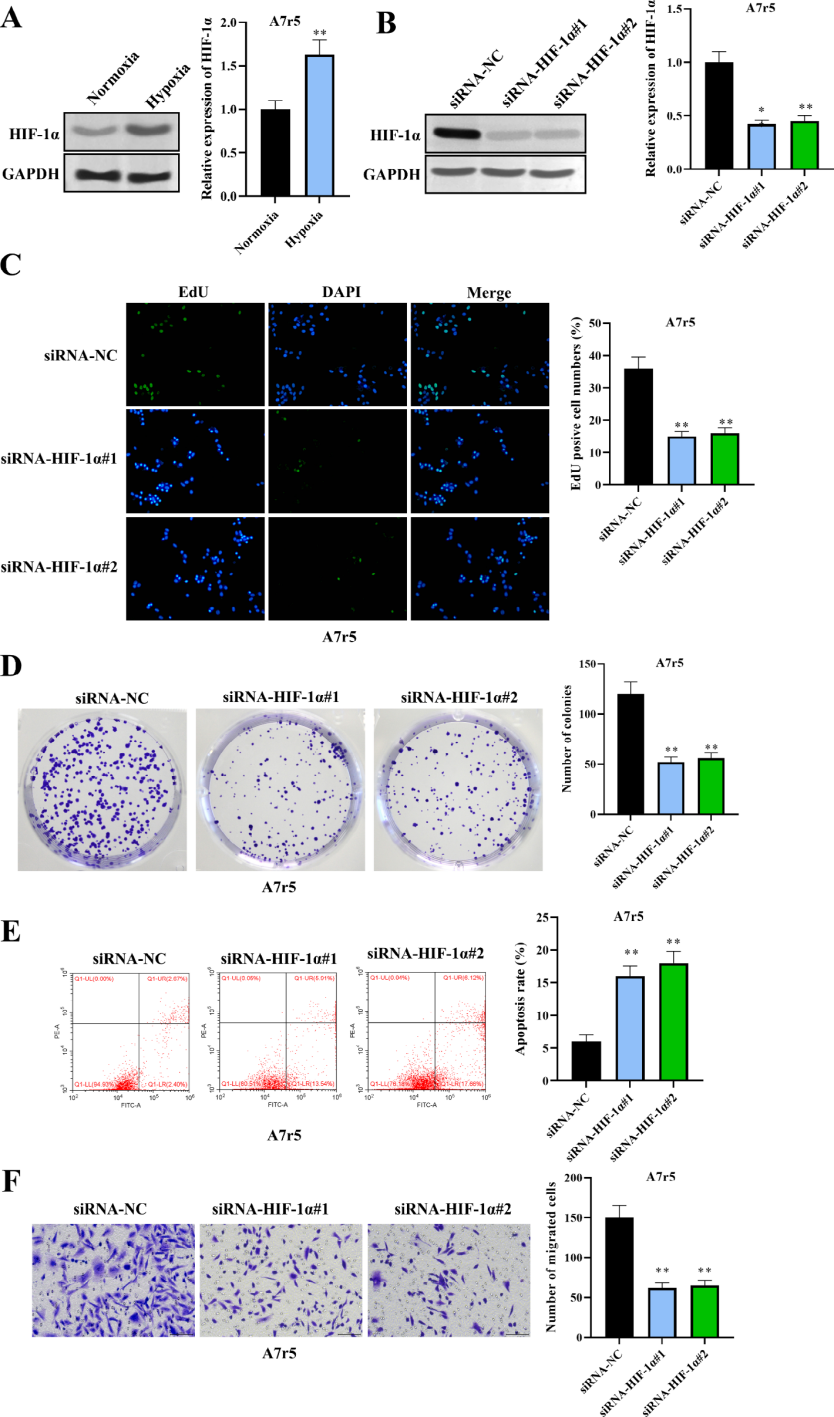
HIF-1α promotes VSMC proliferation and migration while suppressing apoptosis. (**A**) RT‒qPCR and western blot analysis confirmed the hypoxia-induced upregulation of HIF-1α expression in A7r5 cells. (**B**) Transfection with siRNAs targeting HIF-1α effectively reduced HIF-1α expression in hypoxia-treated A7r5 cells, as validated by RT‒qPCR and western blotting. (**C-D**) EdU and colony formation assays demonstrated significantly decreased proliferation in HIF-1α-silenced A7r5 cells compared with that in the hypoxia control group. (**E**) Flow cytometry analysis revealed increased apoptosis in A7r5 cells following HIF-1α knockdown under hypoxic conditions. (**F**) Transwell migration assays revealed a marked reduction in A7r5 cell migration capacity after HIF-1α silencing under hypoxia. *n* = 3. The data are shown as the means ± SDs. (**P < 0.01)

Given the central role of dysregulated VSMC behavior in vascular remodeling (Wang et al. 2023a, b), we examined the effects of HIF-1α on VSMC proliferation, migration, and apoptosis using the A7r5 cell line and human aortic smooth muscle cells (HAoSMCs) under hypoxic conditions. Hypoxia significantly upregulated HIF-1α expression in A7r5 cells and HAoSMCs (Fig. 4A, Figure S1A). Silencing HIF-1α with siRNA effectively reduced HIF-1α levels in hypoxia-exposed cells (Fig. 4B, Figure S1B).

EdU and colony formation assays revealed a marked decrease in the proliferative capacity of HIF-1α-silenced A7r5 cells and HAoSMCs compared with that of the hypoxia control group (Fig. 4C and D, Figure S1C-D). Moreover, flow cytometry analysis revealed increased apoptosis in A7r5 cells and HAoSMCs after HIF-1α knockdown (Fig. 4E, Figure S1E). Consistent with these findings, Transwell migration assays revealed significantly reduced cell migration following HIF-1α silencing (Fig. 4F, Figure S1F).

These findings underscore the critical role of HIF-1α in regulating the key cellular behaviors that contribute to pathological vascular changes.

### HIF-1α modulates VSMC Behavior via activation of the Hippo–YAP pathway

**Fig. 5:**
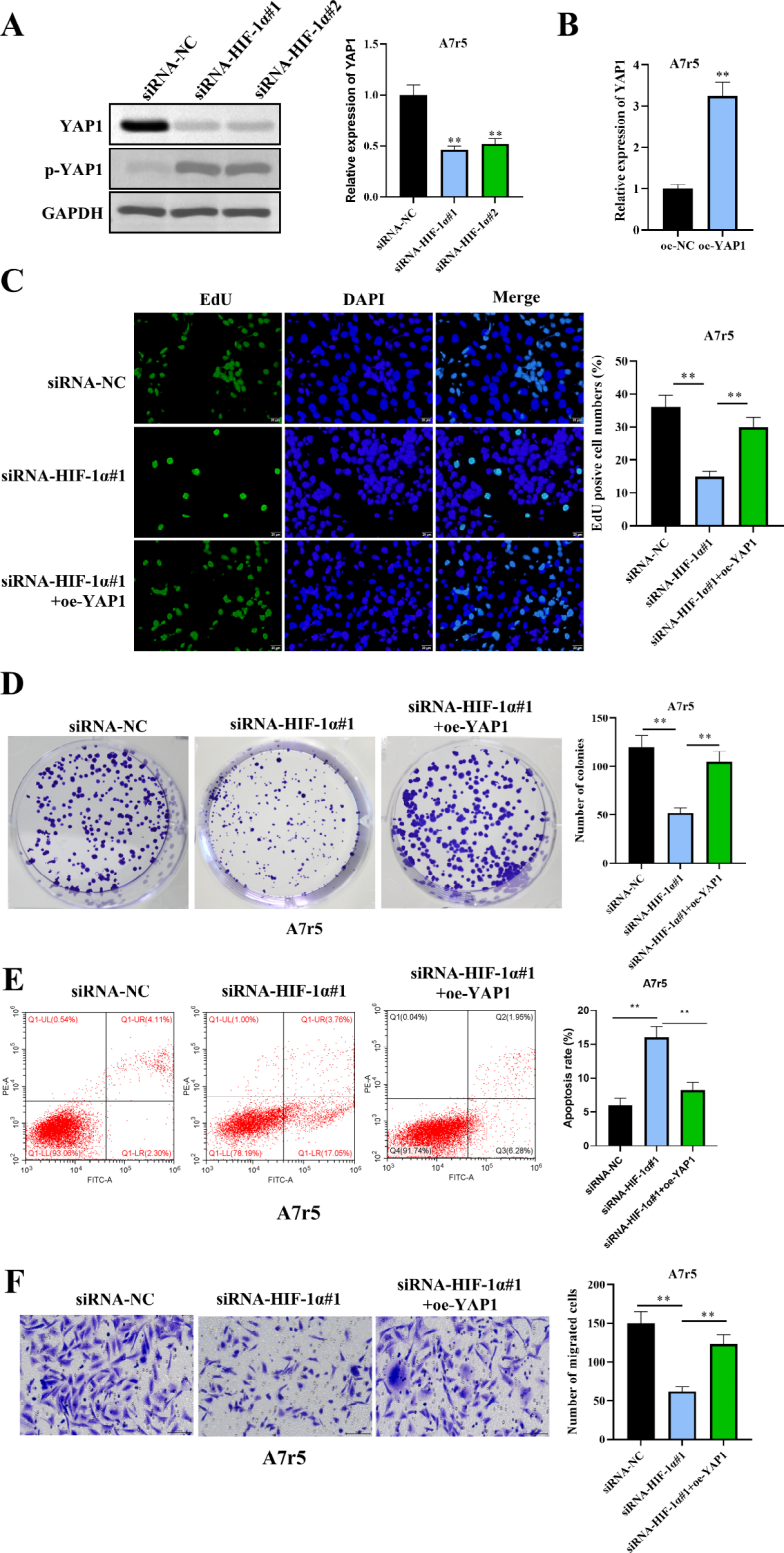
HIF-1α modulates VSMC behavior via Hippo–YAP pathway activation. (**A**) HIF-1α silencing in hypoxia-treated A7r5 cells led to decreased YAP1 expression and increased YAP1 phosphorylation (p-YAP1), indicating Hippo pathway activation, as determined by RT‒qPCR and western blotting. (**B**) RT‒qPCR validation of successful YAP1 overexpression in A7r5 cells under hypoxia. (**C-F**) Rescue experiments: YAP1 overexpression in HIF-1α-silenced A7r5 cells reversed the inhibitory effects on cell proliferation (**C-D**), apoptosis (**E**), and migration (**F**), as demonstrated by EdU, colony formation, flow cytometry, and Transwell assays, respectively. *n* = 3. The data are shown as the means ± SDs. (**P < 0.01)

To elucidate the mechanism by which HIF-1α regulates VSMC function, we explored its interplay with the Hippo–YAP signaling pathway. Notably, HIF-1α silencing resulted in decreased expression of the transcriptional coactivator YAP1, along with increased phosphorylation of YAP1 (p-YAP1), in A7r5 cells and HAoSMCs, indicating pathway inactivation (Fig. 5A, Figure S2A). To confirm the involvement of the Hippo–YAP pathway, we conducted rescue experiments in which YAP1 was overexpressed in A7r5 cells and HAoSMCs (Fig. 5B, Figure S2B). YAP1 overexpression successfully reversed the inhibitory effects of HIF-1α silencing on VSMC proliferation, migration and apoptosis (Fig. 5C and F, Figure S2C-F). These results highlight the complex regulatory network involving HIF-1α and the Hippo–YAP pathway, which orchestrates the cellular dynamics essential for vascular remodeling under hypoxic conditions.

## Discussion

SAS and hypertension coexist and are closely related (Gonzalez-Pliego et al. 2016). SAS-induced oxidative stress is the main cause of hypertension (Dumitrascu et al. 2013). In addition, inflammation is considered copathogenesis of SAS together with hypertension (Wang et al. 2023a, b). As a crucial end product of lipid oxidation, MDA induces tissue damage and is a vital index for evaluating oxidative damage (Tsikas 2017). SOD is a crucial antioxidant enzyme that over clears reactive oxygen species (ROS) (Yang et al. 2022). Hs-CRP is an indicator of systemic inflammation (Moutachakkir et al. 2017), IL-6 can trigger and enhance vascular inflammation and contribute to SAS (Imani et al. 2020), and sE-s are indicators of endothelial dysfunction (Roldan et al. 2003). In the present study, the levels of hs-CRP, IL-6 and sE-s in the serum of SAS-hypertensive rats induced by CIH were elevated, the MDA level was increased, and the SOD level was decreased, suggesting that high levels of inflammation and severe oxidative damage are related to SAS combined with hypertension.

Vascular remodeling is a complicated process involving thickening of the vascular wall, narrowing of the lumen and vascular dysfunction (Wang and Khalil 2018). The imbalance between the proliferation and apoptosis of VSMCs as well as the transition from a contractile phenotype to a synthetic phenotype of VSMCs are crucial factors for vascular remodeling (Yu et al. 2022). During hypertension, the expression of α-SMA, a systolic marker of VSMCs, is decreased (Zhang et al. 2017). The proliferation, migration and synthesis of large amounts of ECM components in VSMCs can also cause vascular remodeling (Zou et al. 2022). TGF-β1 induces ECM deposition along with fibroblast proliferation, stimulates the transformation of fibroblasts into myofibroblasts, and promotes vascular remodeling (Jia et al. 2023). Therefore, α-SMA together with TGF-β1 is a vital indicator of vascular remodeling. In the present study, CIH exposure reduced α-SMA expression but increased TGF-β1 expression in rats. Moreover, histological staining revealed increased collagen deposition and changes consistent with vascular remodeling after exposure to CIH.

Repeated apnea leads to CIH-induced HIF-1α upregulation and increased ROS production, thus promoting the occurrence of hypertension (Guo et al. 2022). In this study, HIF-1α expression increased in rats after CIH exposure. Knockdown of HIF-1α repressed the systolic blood pressure, oxidative stress, inflammation and vascular remodeling stimulated by CIH, which was consistent with the findings of previous studies (Luo et al. 2019).

It is well known that YAP1 can promote VSMC phenotypic modulation and vascular remodeling (Osman et al. 2021; Lin et al. 2018). HIF-1α increases the nuclear localization of YAP while decreasing its phosphorylation (Ma et al. 2017). Hypoxia promotes the binding of YAP to HIF-1α in the nucleus, thereby stabilizing the HIF-1α protein (Zheng et al. 2023). Our research revealed that silencing HIF-1α reduced YAP1 expression but increased p-YAP1 levels. Moreover, overexpression of YAP1 reversed the inhibited proliferation and migration as well as increased apoptosis in HIF-1α-silenced A7r5 cells and HAoSMCs, suggesting that HIF-1α promotes VSMC proliferation by activating the Hippo–YAP pathway, which was in line with the findings of a previous study (Chen et al. 2018).

This study has several limitations. First, only young adult male rats were used, whereas sleep apnea is commonly observed in elderly women in clinical settings. Future studies should incorporate both aging and sex as variables to fully explore the role of HIF-1α and its regulatory mechanisms. Second, while we investigated the role of the Hippo–YAP pathway in hypoxia-exposed cells, further validation of whether HIF-1α exacerbates CIH-induced hypertension through the Hippo–YAP pathway in vivo is necessary.

In conclusion, this study is the first to demonstrate that HIF-1α knockdown alleviates hypertension, inflammation, oxidative stress, and vascular remodeling in a CIH-induced hypertension model. These effects are mediated through the activation of the Hippo–YAP pathway. Our findings suggest that targeting HIF-1α could be a promising therapeutic strategy for managing CIH-related hypertension in SAS patients.

## Electronic supplementary material

Below is the link to the electronic supplementary material.

Supplementary Material 1

## Data Availability

The corresponding author can provide the data that support the findings of this study upon request.
