# Supplementary material for: HIF-1α mediates hypertension and vascular remodeling in sleep apnea via hippo–YAP pathway activation
Source: Mol Med. 2024 Dec 28;30:281. doi: 10.1186/s10020-024-00987-5 (PMC11681631; doi:10.1186/s10020-024-00987-5)
Supplement: Supplementary file 1 — Supplementary Material 1 [file 10020_2024_987_MOESM1_ESM.docx]

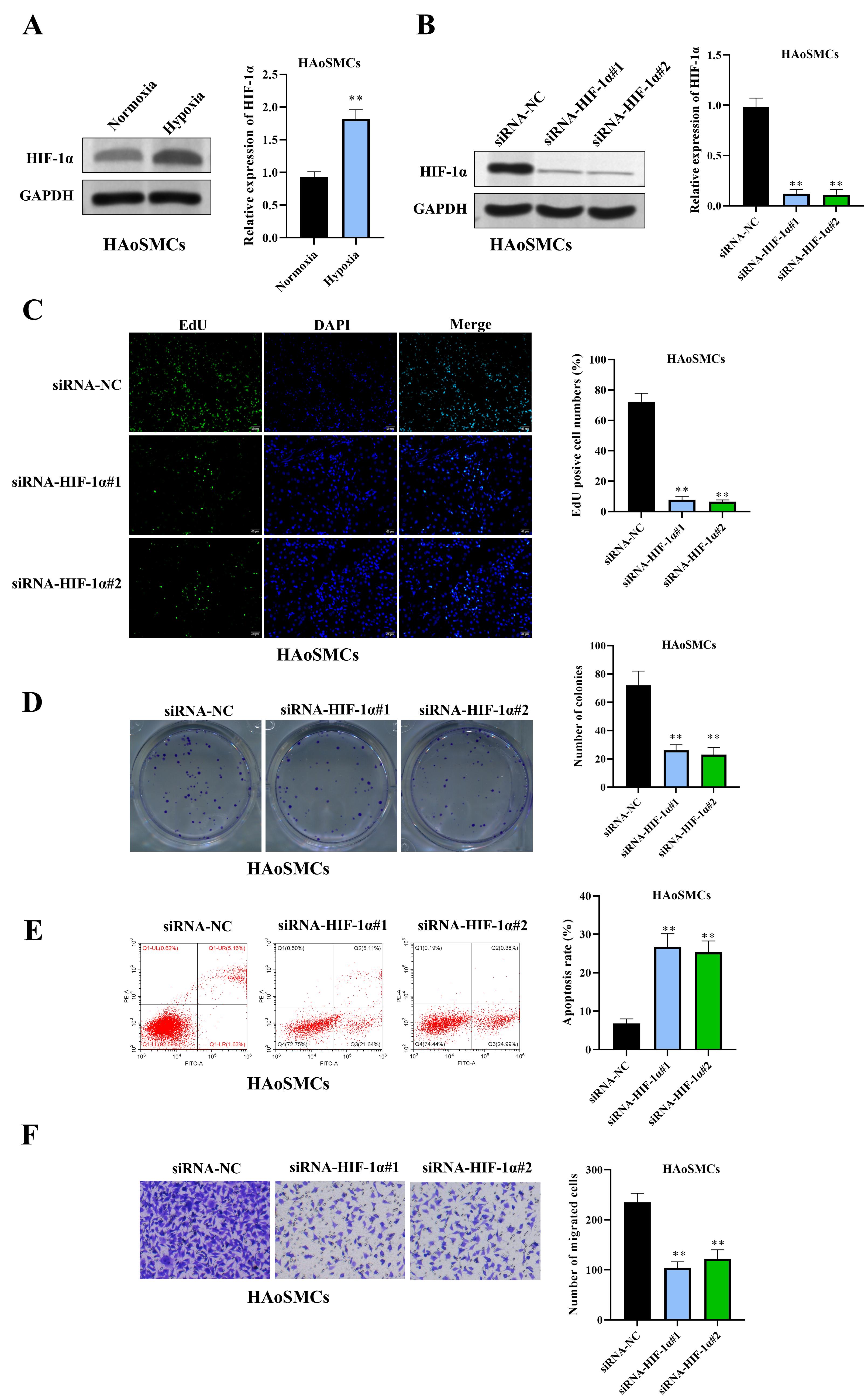


**Figure S1. HIF-1α promotes proliferation and migration and suppresses the apoptosis of HAoSMCs.** (A) Western blot and RT‒qPCR analyses confirmed the hypoxia-induced upregulation of HIF-1α expression in HAoSMCs. (B) Transfection with siRNAs targeting HIF-1α effectively reduced HIF-1α expression in hypoxia-treated HAoSMCs, as validated by western blot and RT‒qPCR analyses. (C-D) EdU and colony formation assays revealed the impact of HIF-1α silencing on the proliferation of HAoSMCs under hypoxic conditions. (E) Flow cytometry analysis revealed increased apoptosis in HAoSMCs following HIF-1α knockdown under hypoxic conditions. (F) Transwell migration assays revealed the migration capacity of HAoSMCs after HIF-1α silencing under hypoxia. n=3. The data are shown as the means ± SDs. (***P*<0.01).


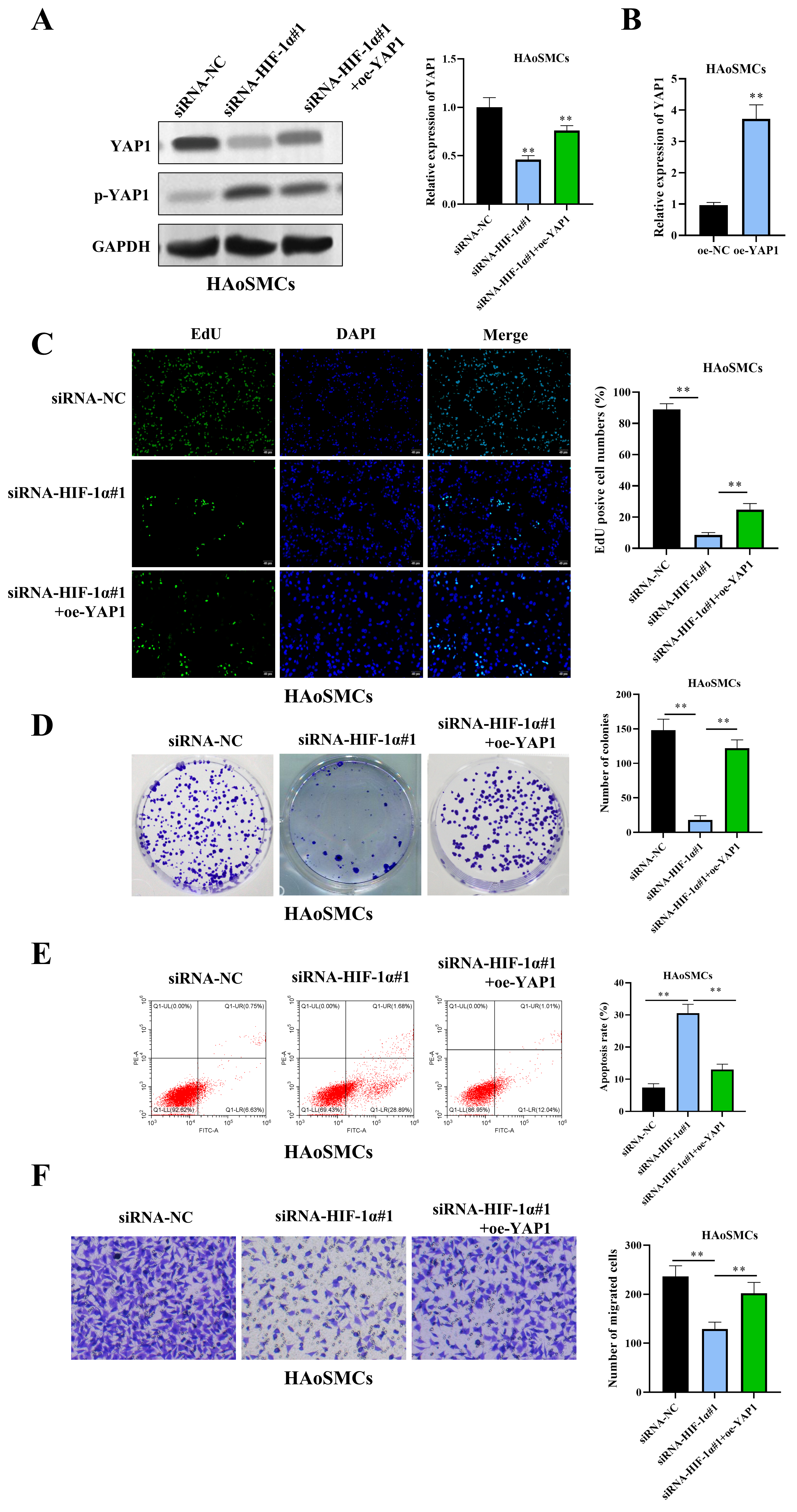


**Figure S2.** **HIF-1α modulates the behavior of HAoSMCs via Hippo–YAP pathway activation.** (A) HIF-1α silencing in hypoxia-exposed HAoSMCs decreased YAP1 expression and increased YAP1 phosphorylation (p-YAP1) levels, as determined by western blotting and RT‒qPCR. (B) RT‒qPCR revealed successful YAP1 overexpression in HAoSMCs exposed to hypoxia. (C-F) Rescue experiments were conducted to evaluate the effects of YAP1 overexpression on the proliferation (C-D), apoptosis (E), and migration (F) of HIF-1α-silenced HAoSMCs via EdU, colony formation, flow cytometry, and Transwell assays, respectively. n=3. The data are shown as the means ± SDs. (***P*<0.01).
